# Prognostic value of brain natriuretic peptide in acute pulmonary embolism

**DOI:** 10.1186/cc6996

**Published:** 2008-08-22

**Authors:** Guillaume Coutance, Olivier Le Page, Ted Lo, Martial Hamon

**Affiliations:** 1 Service des Maladies du Coeur et des Vaisseaux, UF Soins Intensifs Cardiologiques, Centre Hospitalier Universitaire de Caen, Avenue Côte de Nacre, 14033 Caen Cedex, Normandy, France; 2 Service de Chirurgie Cardiaque, Centre Hospitalier Universitaire de Caen, Avenue Côte de Nacre, 14033 Caen Cedex, Normandy, France; 3 Inserm 744, Institut Pasteur de Lille, 1 rue du Professeur Calmette, 59019 Lille cedex, France

## Abstract

**Introduction:**

The relationship between brain natriuretic peptide (BNP) increase in acute pulmonary embolism (PE) and the increase in mortality and morbidity has frequently been suggested in small studies but its global prognostic performance remains largely undefined. We performed a systematic review and meta-analysis of data to examine the prognostic value of elevated BNP for short-term all-cause mortality and serious adverse events.

**Methods:**

The authors reviewed PubMed, BioMed Central, and the Cochrane database and conducted a manual review of article bibliographies. Using a prespecified search strategy, we included a study if it used BNP or N-terminal pro-BNP biomarkers as a diagnostic test in patients with documented PE and if it reported death, the primary endpoint of the meta-analysis, in relation to BNP testing. Studies were excluded if they were performed in patients without certitude of PE or in a subset of patients with cardiogenic shock. Twelve relevant studies involving a total of 868 patients with acute PE at baseline were included in the meta-analysis using a random-effects model.

**Results:**

Elevated BNP levels were significantly associated with short-term all-cause mortality (odds ratio [OR] 6.57, 95% confidence interval [CI] 3.11 to 13.91), with death resulting from PE (OR 6.10, 95% CI 2.58 to 14.25), and with serious adverse events (OR 7.47, 95% CI 4.20 to 13.15). The corresponding positive and negative predictive values for death were 14% (95% CI 11% to 18%) and 99% (95% CI 97% to 100%), respectively.

**Conclusion:**

This meta-analysis indicates that, while elevated BNP levels can help to identify patients with acute PE at high risk of death and adverse outcome events, the high negative predictive value of normal BNP levels is certainly more useful for clinicians to select patients with a likely uneventful follow-up.

## Introduction

Accurate risk stratification in patients with pulmonary embolism (PE) is of first importance in selecting the optimal management strategy for each individual and to potentially improve patient outcome [1-12]. Indeed, in-hospital mortality associated with PE depends on clinical features at admission and increases significantly when right ventricular (RV) dysfunction is documented by echocardiography even in the absence of hemodynamic deterioration [11]. Brain natriuretic peptide (BNP) is a neurohormone secreted from cardiac ventricles in response to ventricular strain. It has been suggested that BNP or N-terminal pro-BNP (NT-proBNP) might be valuable biomarkers for the diagnosis of the RV dysfunction in acute PE and subsequently to predict mortality and serious adverse events (SAEs), especially in patients with initial normal hemodynamic status [12]. However, the magnitude of this progonostic value assessed in a number of small studies remains largely undefined. Therefore, we performed a meta-analysis of studies in patients with acute PE to evaluate the relation between elevated BNP or NT-proBNP levels and clinical outcome.

## Materials and methods

### Study objectives

The primary objective of this meta-analysis was to assess the prognostic value of elevated BNP or NT-proBNP levels to predict short-term mortality (in-hospital or up to 40-day all-cause mortality) in patients with acute PE. The secondary objectives were to evaluate whether BNP increases are associated with short-term mortality resulting from PE (cause-specific mortality) or with SAEs.

### Study endpoints

Total death and death resulting from PE were adjudicated by the authors of the individual studies. Death resulting from PE was related to irreversible RV failure or recurrent PE. SAEs were the composite of death and any of the following adverse outcome events: shock, need for thrombolysis, nonfatal PE recurrence, cardiopulmonary resuscitation, mechanical ventilation, catecholamnine administration, and surgical embolectomy.

### Search strategy

The authors reviewed PubMed, BioMed Central, and the Cochrane database and conducted a manual review of article bibliographies. Unrestricted database searches until March 2008 were performed using the combined medical subject headings for 'BNP', 'pulmonary embolism', 'outcome', 'prognostic', and 'NT-proBNP' with the exploded term 'acute pulmonary embolism'. The retrieved studies were carefully examined to exclude potentially duplicate or overlapping data. Meetings abstracts were excluded as they could not provide adequately detailed data and their results might not be final. Only papers evaluating the role of BNP or NT-proBNP on patient outcomes (death or SAE) were included. Studies were eligible regardless of whether they referred to subjects with small or severe PE.

### Study eligibility

We included a study if (a) it used BNP or NT-proBNP biomarkers as a diagnostic test in patients with documented PE (using a conventional threshold for positivity of the test), (b) it reported death as the primary endpoint of the study and/or SAEs in relation to BNP testing, or (c) it reported deaths and SAEs in absolute numbers for calculation of true-positive (death with BNP increased), false-positive (survival with BNP increased), true-negative (survival with normal BNP level), and false-negative (death with normal BNP level) results or presented sufficiently detailed data for deriving these figures or were provided by the authors when their studies did not report the full data. Studies were excluded if they were performed (a) in patients without certitude of PE, (b) in a subset of patients with cardiogenic shock, or (c) with fewer than 20 enrolled patients as there is a higher risk of invalid results due to selection bias.

### Data extraction

The following information was extracted from each study: first author, year of publication, and journal; study population characteristics, including sample size (number of subjects evaluated with BNP tests and number of patients excluded); number of patients with documented PE; gender; mean age (and standard deviation); relative timing of BNP assessment; technical characteristics of the BNP test and threshold, including type and brand of test used; and rate of short-term death and rate of SAEs as previously defined according to BNP or NT-proBNP tests. Two investigators (GC and MH) performed the data extraction independently. Disagreements were resolved by discussion and consensus. The study was conducted according to MOOSE (Meta-analysis Of Observational Studies in Epidemiology) guidelines [13]. Unlike randomized controlled trials, no generally accepted lists of appropriate quality criteria for observational studies are available. Rather than producing a simple arbitrary quality score, specific quality aspects were used to assess the studies such as control of confounding factors, minimization of selection bias with clear description of inclusion and exclusion criteria, description of the baseline characteristics of the cohort, completeness of follow-up, clear definition of study outcomes, relative timing of the biomarker assessment after patient admission, and whether or not the investigator responsible for BNP measurements was unaware of the patients' baseline characteristics or clinical course.

### Data synthesis and statistical analysis

Categorical variables from individual studies are presented as n/N (number of cases/total number of patients, percentage), and continuous variables are presented as mean values. Measures of odds ratio (OR) and of diagnostic performance are reported as point estimates (with 95% confidence intervals [CIs]). The main analysis was performed on the prognostic value of BNP testing to predict death. Secondary analyses combined the available SAE data to calculate prognostic performance.

By means of true-positive, true-negative, false-positive, and false-negative rates, we computed sensitivity, specificity, positive and negative likelihood ratios, and ORs. While predictive values are well known as measures of diagnostic accuracy, their results may be influenced by the prevalence of disease in tested subjects. The positive likelihood ratio (the ratio between sensitivity and 1 – specificity) provides an estimate of the probability of a positive test in a patient with disease, and the negative likelihood ratio (the ratio between 1 – sensitivity and specificity) gives an estimate of the probability of a negative test among diseased subjects. Both likelihood ratios are roughly independent from prevalence rates, and there is consensus that a positive likelihood ratio of greater than 10 and a negative likelihood ratio of less than 0.1 provide reliable evidence of satisfactory diagnostic performance. While likelihood ratios are the recommended summary statistics for systematic reviews of diagnostic studies, predictive values may also be of interest for clinicians, even if these values vary widely in their dependence on disease prevalence. Such limitations of predictive values notwithstanding, these figures were also computed and reported as exploratory data in this review. Weighted symmetric summary receiver operating characteristic plots, with pertinent areas under the curve, were computed using the Moses-Shapiro-Littenberg method.

We computed all statistics for individual studies, then combined them using a random-effects model, weighting each point estimate by the inverse of the sum of its variance and the between-study variance. Between-study statistical heterogeneity was also assessed using the Cochran Q chi-square test and the I^2 ^test. Separate analyses were performed on studies with BNP and proBNP assessments. Publication bias was assessed visually by examination of funnel plots. Statistical computations were performed with SPSS 11.0 (SPSS Inc., Chicago, IL, USA), Meta-DiSc [14], and Review Manager 4.2 [15], and significance testing was at the two-tailed 0.05 level.

## Results

### Description of studies

**Figure 1:**
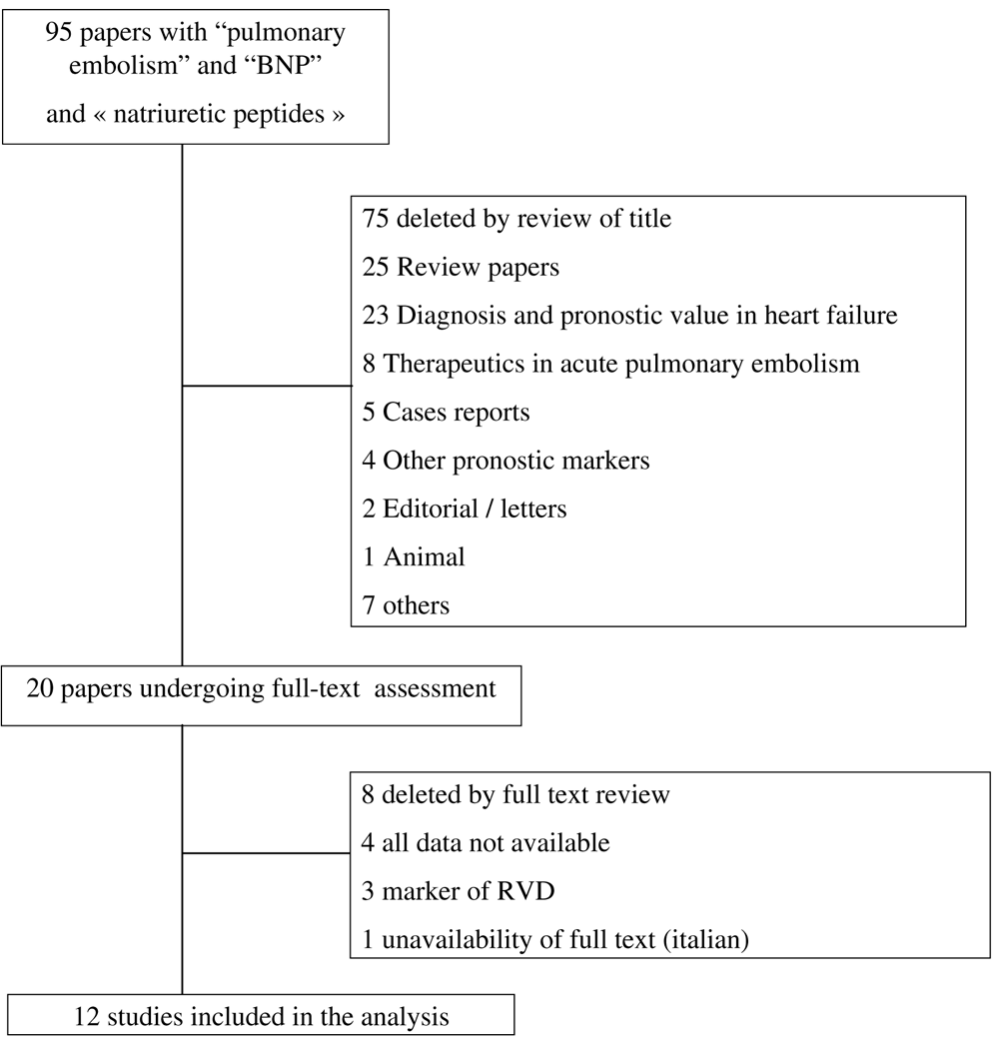
Flow diagram for study selection. BNP, brain natriuretic peptide; RVD, right ventricular dysfunction.

Overall, 12 studies [1-12] were included in this analysis after study selection described in Figure 1. Baseline characteristics of included studies are shown in Table 1. All studies were prospective studies with BNP or NT-proBNP assessments measured in the vast majority of cases at admission. Demographic features (age and gender) were homogenous across studies, and almost all patients had a confirmed diagnosis of PE. RV dysfunction according to BNP or NT-proBNP levels was reported in eight studies. Overall, RV dysfunction was present in 76.2% of cases.

**Table 1:** Characteristics of included studies

Reference	Study design	Patients, number	Hemodynamic instability (number)	Timing of BNP sampling	Thrombolysis, number	Age, years	Male, percentage	Follow-up	CHF, number
Kucher, *et al*. [1] (2003)	Prosp	73	Yes	Admission (<4 hours)	10	61 ± 18	59	In hosp	NA
Ten Wolde, *et al*. [2] (2003)	Prosp	110	Excl	Admission	NA	58 ± 18	NK	3 months	NA
Pieralli, *et al*. [3] (2006)	Prosp	61	Excl	Admission (<1 hour)	6	75 ± 14	26	In hosp	Excl
Krüger, *et al*. [4] (2004)	Prosp	42	Yes (10)	Admission	22	57 ± 17	64	In hosp	8 Excl
Tulevski, *et al*. [5] (2006)	Prosp	28	Excl	Admission (<1 hour)	NA	53 ± 18	43	90 days	Excl
Logeart, *et al*. [6] (2007)	Prosp	67	Excl	Admission	6	64 ± 16	60	In hosp	Excl
Ray, *et al*. [7] (2006)	Prosp	51	NA	Admission	0	79 ± 10	NA	In hosp	NA
Pruszczyk, *et al*. [8] (2003)	Prosp	79	Yes (9)	Admission	8	63 ± 17	37	In hosp	NA
Kucher, *et al*. [9] (2003)	Prosp	73	Yes (14)	Admission (<4 hours)	10	61 ± 18	59	In hosp	6
Kostrubiec, *et al*. [10] (2005)	Prosp	100	Excl	Admission	5	62 ± 18	35	40 days	17
Binder, *et al*. [11] (2005)	Prosp	124	Yes (9)	Admissionand at 4, 8, and 24 hours	12	60 ± 18	40	In hosp	NA
Maziere, *et al*. [12] (2007)	Prosp	60	Excl	Admission	NA	73 ± 14	40	In hosp	20

Values are presented as mean ± standard deviation when appropriate. BNP, brain natriuretic peptide; CHF, congestive heart failure; Excl, excluded; In hosp, in hospital; NA, not applicable; Prosp, prospective.

### Brain natriuretic peptides assays

As shown in Table 2, different assays for BNP or NT-proBNP measurements were used throughout the studies, with different cutoff points for abnormal levels. In most of the studies, the cut points for BNP assays were not predefined but derived from receiver operating characteristic curve construction to determine the best threshold able to predict complicated PE. One study reported outcomes for two assays using BNP and NT-proBNP levels [1,9]. We performed separate analyses including both cohorts or excluding one or the other with similar results.

**Table 2:** Characteristics of brain natriuretic peptide (BNP) and N-terminal pro-BNP assays

Reference	BNP/NT-proBNP	Assay	Manufacturer	Kind of assay	Cutoff, pg/mL	Elevated BNP, percentage
Kucher, *et al*. [1]	BNP	Fluorescence immunoassay	Biosite (San Diego, USA)	Quantitative	90	43.8
Ten Wolde, *et al*. [2]	BNP	Immunoradiometric assay	Shionoria (Osaka, Japan)	Quantitative	21.7	33
Pieralli, *et al*. [3]	BNP	Fluorescence immunoassay	Biosite	Quantitative	527	67
Krüger, *et al*. [4]	BNP	Immunofluorometric assay	Biosite	Quantitative	90	40
Tulevski, *et al*. [5]	BNP	Immunoradiometric assay	Shionoria	Quantitative	10	50
Logeart, *et al*. [6]	BNP	Fluorescence immunoassay	Biosite	Quantitative	100	70
Ray, *et al*. [7]	BNP	Fluorescence immunoassay	BioMérieux (Marcy l'Etoile, France)	Quantitative	200	43
Pruszczyk, *et al*. [8]	NT-proBNP	ECLIA	Roche (Basel, Switzerland)	Quantitative	NA	83.5
Kucher, *et al*. [9]	NT-proBNP	ECLIA	Roche	Quantitative	500	57
Kostrubiec, *et al*. [10]	NT-proBNP	ECLIA	Roche	Quantitative	600	39
Binder, *et al*. [11]	NT-proBNP	ECLIA	Roche	Quantitative	1,000	54
Maziere, *et al*. [12]	NT-proBNP	ECLIA	Roche	Quantitative	1,000	43

ECLIA, enhanced chemiluminescence immunoassay; NA, not applicable; NT-proBNP, N-terminal pro-brain natriuretic peptide. Triage BNP test is manufactured by Biosite (San Diego, USA).

### Outcome measures

#### Death

**Figure 2:**
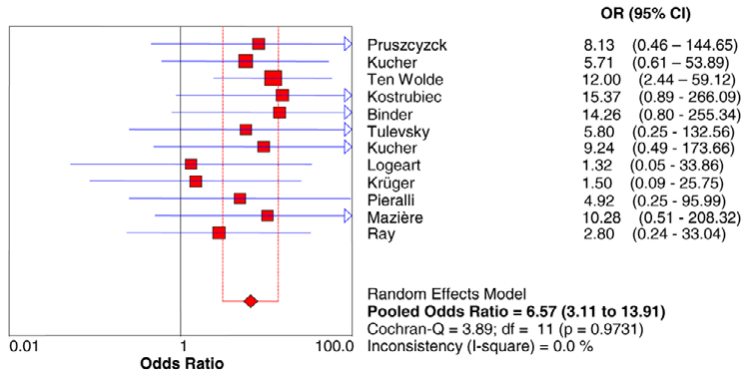
Odds ratio (OR) for death based on elevated or normal brain natriuretic peptide levels in acute pulmonary embolism. CI, confidence interval; df, degrees of freedom.

**Figure 3:**
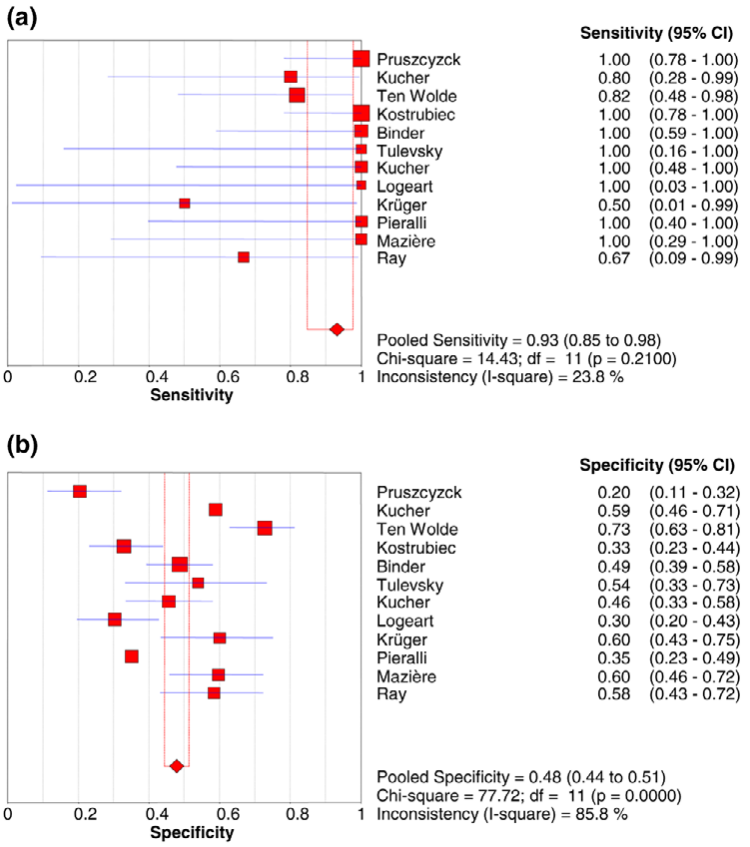
Pooled sensitivities **(a) **and specificities **(b) **of elevated brain natriuretic peptide levels to predict short-term death in acute pulmonary embolism. CI, confidence interval; df, degrees of freedom.

**Figure 4:**
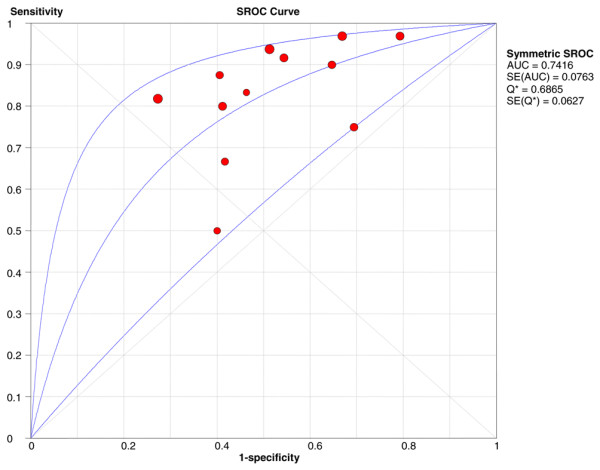
Plot of symmetric summary receiver operator characteristic (SROC) of elevated brain natriuretic peptide levels to predict short-term death. The receiver operator characteristic curve provides a graphical display of diagnostic accuracy by plotting 1 – specificity in the horizontal axis and sensitivity in the vertical axis. The pertinent area under the curve (AUC) and Q* statistic (the point where sensitivity and specificity are maximal), both with standard errors (SEs), are also included.

Data on death, the primary endpoint of the present meta-analysis, was reported in 12 studies including 868 patients. Among these patients, 482 (55.5%) had BNP increased and 68 died (14.1%; 95% CI 11.1% to 17.5%) compared with 386 (44.5%) with normal BNP levels with 5 deaths observed (1.3%; 95% CI 0.04% to 3.0%). Increased BNP or NT-proBNP levels were associated with a higher risk of short-term death (OR 6.57, 95% CI 3.11 to 13.91) with no heterogeneity observed (Figure 2). The results were consistent for either BNP (OR 5.06, 95% CI 2.02 to 12.65) [1-7] or proBNP (OR 11.15, 95% CI 3.03 to 40.97) [8-12] studies. The association between elevated BNP or NT-proBNP levels and death was confirmed also after substituting 0.5 for 0 in the random-effects model (OR 6.20, 95% CI 2.92 to 13.17). The sensitivity and specificity of increased BNP or NT-proBNP levels to predict death were 0.93 (95% CI 0.85 to 0.98) and 0.48 (95% CI 0.44 to 0.51), respectively (Figure 3 and Table 3), with the symmetric summary receiver operator characteristic curve shown in Figure 4. The corresponding positive and negative likelihood ratios are given in Table 3 as well as positive and negative predictive values. Interestingly, the negative predictive value was found to be very high: 99% (95% CI 97% to 100%).

**Table 3:** Pooled summary results of the prognostic value of elevated brain natriuretic peptide in acute pulmonary embolism

Endpoints	OR (95% CI)	Sensitivity (95% CI)	Specificity (95% CI)	LR+ (95% CI)	LR- (95% CI)	PPV (95% CI)	NPV (95% CI)
Short-term death (12 studies, 868 patients)	6.57 (3.11–13.91)	0.93 (0.85–0.98)	0.48 (0.44–0.51)	1.64 (1.39–1.94)	0.34 (0.19–0.61)	0.14 (0.11–0.18)	0.99 (0.97–1.00)
Death resulting from PE (10 studies, 684 patients)	6.10 (2.58–14.25)	0.92 (0.81–0.98)	0.52 (0.48–0.56)	1.76 (1.33–2.34)	0.37 (0.19–0.71)	0.13 (0.10–0.17)	0.99 (0.97–1.00)
Serious adverse events (9 studies, 688 patients)	7.47 (4.2–13.15)	0.89 (0.83–0.93)	0.48 (0.44–0.52)	1.70 (1.44–2.01)	0.28 (0.17–0.48)	0.33 (0.29–0.38)	0.94 (0.90–0.96)

CI, confidence interval; LR+, positive likelihood ratio; LR-, negative likelihood ratio; NPV, negative predictive value; OR, odds ratio; PE, pulmonary embolism; PPV, positive predictive value.

#### Cause-specific death resulting from pulmonary embolism

Ten studies reported on deaths resulting from PE in 684 patients. The rates of death resulting from PE were 13.3% (47 of 353; 95% CI 9.95% to 17.31%) in patients with BNP increased and 1.2% (4 of 331; 95% CI 0.33% to 3.07%) in patients without BNP increased. Elevated BNP or NT-proBNP levels were associated with higher risk of death resulting from PE (OR 6.10, 95% CI 2.58 to 14.25) (Table 3) with no heterogeneity found. Pooled summary results of diagnostic performance are listed in Table 3 with a remarkably high negative predictive value of 99% (95% CI 97% to 100%).

#### Serious adverse events

Nine studies reported on the occurrence of SAEs. The rates of SAE were 33.2% (138 of 415; 95% CI 28.73% to 38.01%) and 6.2% (17 of 273; 95% CI 3.67% to 9.78%) in patients with and without elevated BNP levels, respectively. Elevated BNP or NT-proBNP levels were associated with higher risk of SAE (OR 7.47, 95% CI 4.2 to 13.5) with no heterogeneity observed. Pooled summary results of diagnostic performance are listed in Table 3.

## Discussion

This meta-analysis indicates that elevated BNP or NT-proBNP levels can help to identify patients with acute PE at high risk of short-term death and adverse outcome events. However, while sensitivity of this biomarker is high to detect patients at risk of death or of SAEs, the specificity remains low. In keeping with these results, however, the high negative predictive value might be useful for clinicians to select patients with a likely uneventful follow-up. Indeed, accurate risk stratification in patients with PE is of first importance in selecting the optimal management strategy for each individual and to potentially improve patient outcome. Acute PE is frequently accompanied not only by dyspnoae, but also by RV dysfunction leading to BNP release. In hemodynamically stable patients, RV dysfunction as observed by echocardiography has been shown to be able to identify patients with poor outcomes who might require more aggressive treatment like thrombolysis [11]. The availability of biomarkers like BNP or NT-proBNP able to identify RV dysfunction patients early and to contribute to risk stratification is potentially important, especially when echocardiography assessment is not available. In the present meta-analysis, we confirm that BNP or NT-proBNP levels identify patients at higher risk of poor outcome frequently with RV dysfunction, but related to its low specificity, its positive predictive value remains very limited. The BNP or NT-proBNP assessments should become part of the risk evaluation among selected individuals with acute PE but need to be combined with other independent predictors for optimal risk stratification in future studies including troponins and echocardiography, especially for testing the possible benefits of early thrombolysis in the intermediate-risk patient group [11].

The prognostic value of BNP or NT-proBNP was consistent in all studies included, regardless of the specific assay used. Time interval between the acute PE event and BNP measurement was performed frequently at admission but without details about when the symptoms evoking PE started. BNP levels may not correlate well with cardiovascular outcomes in some patients with PE of acute onset because of the obligatory delay in BNP mRNA upregulation and subsequent protein release in the serum. Indeed, it takes several hours for the BNP levels to increase after the onset of acute myocardial stretch. This issue is important for risk stratification and for guiding decision making, and a note of caution is mandatory until longitudinal studies with BNP assessments have been performed.

### Limitations

The major limitation of the present analysis is our inability to determine the exact incremental value of BNP assessment over and in combination with other conventional risk factors or troponin measurement [16] because individual data were not available to us. Therefore, our pooled estimates of prognostic performance are not adjusted for conventional risk factors such as age, gender, hypertension, or prior history of heart failure or of cancer. However, most of the included studies have performed mutivariate analyses confirming the increased risk of death and SAEs in patients with elevated BNP or NT-proBNP levels. Surprisingly, only small differences between adjusted and nonadjusted estimates were found. In fact, the OR appeared greater after adjustment in most studies, suggesting that our estimates may be conservative and may slightly underestimate the true risk increase of adverse outcomes associated with elevated BNP or NT-proBNP levels.

The higher risk of SAE in PE patients with elevated BNP or NT-proBNP levels requires a note of caution given that this endpoint was the aggregate of many outcomes (including death, shock, need for thrombolysis, nonfatal PE recurrence, cardiopulmonary resuscitation, mechanical ventilation, catecholamnine administration, and surgical embolectomy), rendering its interpretation quite challenging.

We should also acknowledge that most studies did not report complete data concerning the timing of BNP and NT-proBNP measurements in relation to the occurrence of acute PE. In this perspective, serial biomarker assessment at least during the first 24 hours after admission for acute PE should be encouraged in future clinical research. Furthermore, all the cutoff concentrations for BNP or NT-proBNP used as prognostic values were defined retrospectively and with wide variations across studies. Therefore, a prospective validation of predefined BNP cutoff is urgently required in a large multicenter study to confirm its prognostic value.

## Conclusion

This meta-analysis indicates that elevated BNP levels can identify patients with acute PE at high risk of short-term death and adverse outcome events. However, while BNP measurements might become part of the risk stratification in PE, its positive predictive value alone remains low and its high negative predictive value is certainly more useful to identify patients with a likely favorable outcome. Whether serial BNP level assessment within the first 24 hours will facilitate risk stratification of patients with PE and subsequently patient management through less aggressive treatment of those with normal BNP levels would need to be tested in future studies.

## Key messages

• Elevated brain natriuretic peptide (BNP) levels can help to identify patients with acute pulmonary embolism at high risk of short-term death and adverse outcome events.

• Although elevated BNP levels have a high sensitivity to detect patients at risk of death, the specificity is low.

• The positive predictive value of elevated BNP levels alone remains low and its high negative predictive value is more useful to identify individuals with a likely favorable outcome.

## Abbreviations

BNP = brain natriuretic peptide; CI = confidence interval; NT-proBNP = N-terminal pro-brain natriuretic peptide; OR = odds ratio; PE = pulmonary embolism; RV = right ventricular; SAE = serious adverse event.

## Competing interests

The authors declare that they have no competing interests.

## Authors' contributions

GC helped to design the study and review the literature. MH helped to design the study and review the literature and performed the statistical analysis. All authors contributed substantially in preparing the manuscript and participated actively in writing the discussion. All authors read and approved the final manuscript.
